# Educational Homogamy Lowers the Odds of Reproductive Failure

**DOI:** 10.1371/journal.pone.0022330

**Published:** 2011-07-26

**Authors:** Susanne Huber, Martin Fieder

**Affiliations:** 1 Department of Anthropology, University of Vienna, Vienna, Austria; 2 Research Institute of Wildlife Ecology, University of Veterinary Medicine Vienna, Vienna, Austria; Tel Aviv University, Israel

## Abstract

Assortative mating based on education is a common phenomenon. We investigated whether it affected parameters of reproductive performance such as childlessness, offspring number and age at first marriage. On the basis of the US census from 1980 (n = 670,631 married US couples), we find that the proportion of childless individuals is usually minimal in women married to a husband of the same educational level. This holds particularly true in the highest and the lowest educated women. Educational homogamy is also associated with a lower average age at first marriage. No obvious effect of educational homogamy on a woman's average offspring number is found, where mean offspring number generally increases both with decreasing woman's and decreasing husband's educational attainment. We conclude that educational homogamy reduces the likelihood of reproductive failure.

## Introduction

Assortative mating, i.e. mating based on similarity more frequently than expected by chance, is a common phenomenon. Similarity between spouses has been reported for various characteristics such as, for instance, age, education, socioeconomic status or physical traits [1]–[5]. Assortative mating may occur due to the higher chances of meeting and interacting with individuals of similar characteristics in common school-, work- or living-environments [6]. In addition, homogamous mating may be advantageous in terms of increasing marriage stability [6]–[8]. From an evolutionary point of view, assortative mating may also carry advantages because it may increase the degree of genetic relatedness between the spouses, thus promoting cooperation and increase inclusive fitness [9].

Even though educational homogamy is a widespread phenomenon [2], [4], [10]–[11], little is known whether educational homogamy affects reproductive success. We therefore investigated on the basis of US census data from year 1980, whether educational homogamy is associated with parameters of women's lifetime reproductive success, i.e. childlessness, offspring number and age at first marriage.

## Methods

We used the 5% US census from year 1980 provided by IPUMS US (Minnesota Population Center. *Integrated Public Use Microdata Series – International: Version 4.0*. Minneapolis: University of Minnesota, 2008), to investigate the relationship between educational homogamy (i.e. both spouses are within the same educational category) and parameters of reproductive performance of women (no reproductive data were available for men). We restricted our analyses to women aged from 46 to 65 years because we were interested in lifetime reproductive success. We further restricted our analyses to women who are still in their first marriage and whose husband lives in the same household, totaling 670,631 married US couples. A woman's husband was associated by the spouse location variable in the woman's record, indicating the spouse's serial number within a household (this association has been done by IPUMS). So we were only able to associate a husband to a woman if both spouses were present in the household.

We used the variable “Educational attainment, international recode [general version]” as a measure of educational attainment: 1 =  less than primary completed; 2 =  primary completed; 3 =  secondary completed; 4 =  university completed. All calculations were carried out on the basis of each possible educational combination between a woman and her husband: W1/M1 indicating that both woman and her husband are in the lowest educational category 1, W1/M2 indicating that the woman is in educational category 1 but her husband in educational category 2; up to W4/M4 indicating that both woman and her husband are in the highest educational category 4. In total we obtained 16 educational combinations: W1/M1; W1/M2; W1/M3; W1/M4; W2/M1; W2/M2, W2/M3; W2/M4; W3/M1; W3/M2; W3/M3; W3/M4; W4/M1; W4/M2; W4/M3; W4/M4.

For each educational combination, we calculated the percentage of childless women, women's mean number of biological children, and women's mean age at marriage (which corresponds to age at first marriage in this sample of women still in their first marriage). We further calculated the frequency of each educational combination. Separately for each woman's educational category, we tested with Chi^2^-test whether the percentage of childlessness, and with ANOVA whether mean offspring number and mean age at marriage differed among educational combinations. The same analyses were performed to analyze differences between homogamous (W1/M1, W2/M2, W3/M3, W4/M4) and heterogamous (all other combinations) couples. In addition, we performed separately for each woman's educational category, a linear mixed model on square root transformed number of children (as number of children is skewed) with husband's educational category and woman's current age as fixed factors, woman's age at marriage as a covariate, and ethnicity (encoded in 10 categories: White, Black, American Indian, Chinese, Japanese, Korean, Vietnamese, Filipino, Indian, Other Asian, other) as random factor. We further performed, again separately for each woman's educational category, a logistic regression of husband's educational category, women's current age, woman's age at marriage, and woman's ethnicity on woman's childlessness (encoded as 0 =  childless, 1 =  at least one child). For reasons of clarity, we do not show the estimates for ethnicity in the results.

## Results

### Childlessness

**Figure 1:**
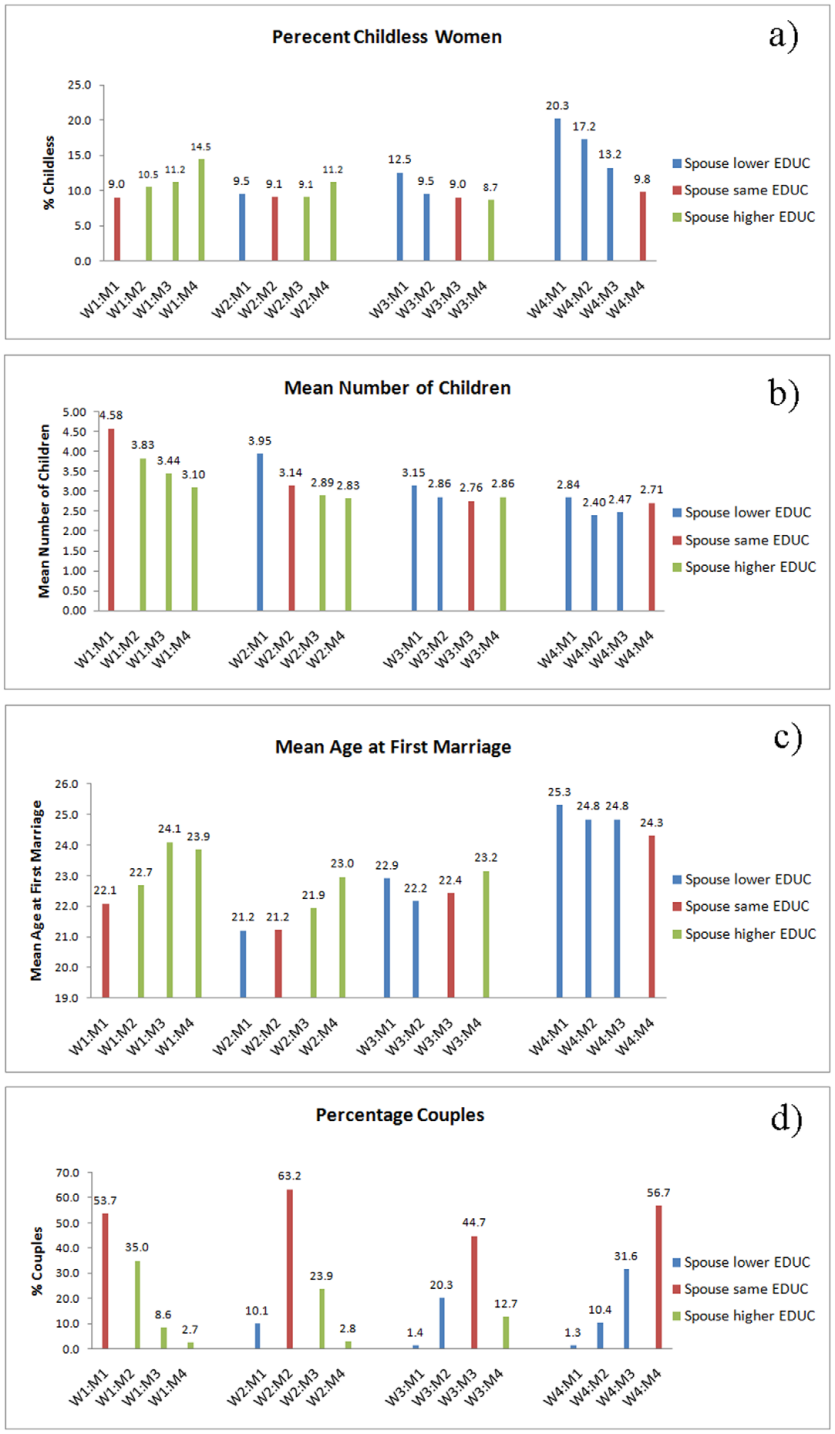
Educational homogamy and parameters of reproduction. (a) Percentage of childless women, (b) woman's mean offspring number, (c) woman's mean age at first marriage, and (d) percentage couples, calculated for each combination of woman's educational category W1 through W4 and husband's educational category M1 through M4 (W1, M1, less than primary completed; W2, M2, primary completed; W3, M3, secondary completed; W4, M4, university completed). Blue bars: wife is higher educated than husband; red bars: both spouses have the same level of education; green bars: husband is higher educated than wife.

We find that the proportion of childless individuals is usually minimal in women married to a husband of the same educational level (Figure 1a). This holds particularly true in the highest and the lowest educated women. The percentage of childless individuals increases with increasing husband's education in the lowest educated women, but decreases with increasing husband's education in the highest educated women. The differences are less pronounced in women of educational category 2, where the least percentage of childlessness is found in women married to an equally or one level higher educated husband. In women of educational category 3, the proportion of childless individuals increases with decreasing husband's education, the lowest percentage found in women married to a husband of educational category 4 (Figure 1a). The differences in the percentage of childless individuals are significant among the educational combinations per woman's educational category as well as between homogamous and heterogamous couples (Table 1).

**Table 1:** Differences in the percentage of childlessness, mean offspring number, and mean age at marriage tested among the education combinations per woman's educational level as well as between homogamous and heterogamous combinations.

	% Childlessness^1^		Offspring Number^2^		Age at Marriage^2^		n
	Chi^2^	P	F	P	F	P	
W1/M1 vs. W1/M2 vs. W1/M3 vs. W1/M4	27.841	<0.001	113.257	<0.001	43.770	<0.001	23287
W2/M1 vs. W2/M2 vs. W2/M3 vs. W2/M4	28.467	<0.001	541.008	<0.001	279.690	<0.001	208580
W3/M1 vs. W3/M2 vs. W3/M3 vs. W3/M4	102.593	<0.001	58.098	<0.001	498.512	<0.001	373949
W4/M1 vs. W4/M2 vs. W4/M3 vs. W4/M4	359.908	<0.001	165.006	<0.001	51.903	<0.001	64815
homogamous vs. heterogamous	78.229	<0.001	2.926	0.087	574.602	<0.001	670631

1 Chi^2^-test, ^2^ANOVA.

In a logistic regression of husband's education, and woman's age, age at marriage and ethnicity (not shown) on women's childlessness (endcoded as 0 =  childless, 1 =  at least one child), in lower educated women, regression coefficients (reference: women married to a husband of educational category 4) increase with decreasing husband's educational level. Again, this indicates that childlessness is more prevalent if a lower educated woman is married to a higher educated than to a lower educated husband (Table 2). Whereas regression coefficients decrease with decreasing husband's educational level in higher educated women, indicating higher chances of childlessness if a higher educated women is married to a lower educated than to a higher educated husband. Regression coefficients of age and age at marriage are always negative, indicating that in this sample of 46 to 65 year old women, frequency of childlessness is higher in older women and in women married at a higher age (Table 2).

**Table 2:** Logistic regression on childlessness (encoded as 0 =  childless, 1 =  at least one child) of woman's age, age at marriage, and ethnicity (not shown) as well as her husband's educational attainment, separately for women of educational category 1 (less than primary completed), educational category 2 (primary completed), educational category 3 (secondary completed), and educational category 4 (university completed).

	Woman's Education 1	Woman's Education 2	Woman's Education 3	Woman’s Education 4
	B (SE)			
Constant	6.300 (0.379)***	6.977 (0.218)***	7.575 (0.235)***	7.699 (0.525)***
Husband’s Education (reference: 4)				
1	0.603 (0.148)***	0.205 (0.055)***	−0.256 (0.051)***	−0.414 (0.140)**
2	0.454 (0.150)**	0.130 (0.049)**	−0.159 (0.019)***	−0.447 (0.049)***
3	0.404 (0.165)*	0.145 (0.050)**	−0.099 (0.017)***	−0.212 (0.030)***
Woman’s Age	−0.037 (0.004)***	−0.037 (0.001)***	−0.027 (0.001)***	−0.012 (0.002)***
Woman’s Age at Marriage	−0.078 (0.002)***	−0.100 (0.001)***	−0.129 (0.001)***	−0.154 (0.002)***
Nagelkerke R^2^	0.132	0.121	0.138	0.193

*p<0.05.

**p<0.01.

***p<0.001.

### Mean Number of Children

There is no obvious effect of educational homogamy on a woman’s average offspring number (Figure. 1b). Even though differences in mean offspring number are significant among the educational combinations per woman’s educational level, mean number of offspring is not significantly different between homogamous and heterogamous couples (Table 1). Generally, mean offspring number increases both with decreasing woman’s and decreasing husband’s educational attainment. In women of educational category 3 and 4, however, women married to the highest educated husbands have, on average, the same number or even more offspring than those married to a husband of educational level 2 or 3 (Figure. 1b). Similarly, in a linear mixed model using a woman’s [transformed] number of children as dependent variable, her age and age at marriage, as well as her husband’s educational attainment as fixed factors and woman’s ethnicity as random factor (not shown), estimates (reference: women married to a husband of educational category 4) increase with decreasing husband’s education level in lower educated women. This indicates higher average offspring number in lower educated woman married to a lower educated than to a higher educated husband (Table 3). In higher educated women, estimates are lowest if they are married to a husband of medium education, indicating that women married either to a husband' of very low or very high education have on average more offspring than those married to a medium educated husband. All estimates of age and age at marriage are negative, indicating that in this sample of 46 to 65 year old women, average offspring number is higher in the younger women and those married at younger age (Table 3).

**Table 3:** Linear mixed model using a woman's [transformed] number of children as dependent variable, her age and age at marriage, as well as her husband's educational attainment as fixed factors, and ethnicity as random factor (not shown), separately for women of educational category 1 (less than primary completed), educational category 2 (primary completed), educational category 3 (secondary completed), and educational category 4 (university completed).

	Woman's Education 1	Woman's Education 2	Woman's Education 3	Woman's Education 4
	Estimate (SE)			
Constant	3.118 (0.097)***	3.272 (0.062)***	3.168 (0.047)***	2.868 (0.055)***
Husband's Education (reference: 4)				
1	0.327 (0.041)***	0.179 (0.011)***	−0.0002 (0.009)	−0.014 (0.028)
2	0.173(0.042)***	0.039 (0.010)***	−0.045 (0.003)***	−0.117 (0.010)***
3	0.107 (0.045)*	0.0004 (0.011)	−0.056 (0.003)***	−0.072 (0.005)***
Woman's Age	−0.011 (0.001)***	−0.013 (0.0003)***	−0.011 (0.0002)***	−0.005 (0.0004)***
Woman's Age at Marriage	−0.035 (0.001)***	−0.035 (0.0003)***	−0.037 (0.0002)***	−0.043 (0.0005)***

*p<0.05.

**p<0.01.

***p<0.001.

### Age at first marriage

Educational homogamy is associated with a lower average age at first marriage (Figure. 1c). In the lowest educated women, mean age at marriage increases with increasing husband's education, whereas it decreases with increasing husband's education in the highest educated women. In women of educational level 2, the lowest average age at marriage is found in those married to a husband of educational level 1 or 2. Only in women of educational level 3, mean age at marriage is lowest if they are married to a husband of educational level 2, followed by husbands of educational level 3 (Figure. 1c). The differences in mean age at marriage are significant among the educational combinations per woman's educational category as well as between homogamous and heterogamous couples (Table 1).

### Proportion of homogamy

Educational homogamy is common in our sample. Irrespective of woman's educational level, we find that the proportion of marriages is highest if both spouses attained the same level of education (Figure. 1 d).

## Discussion

There is a high degree of assortative mating on the basis of educational level in our sample: dependent upon women's education, between 44.7% and 63.2% of couples show educational homogamy. Assortative mating has been reported for a great variety of traits such as for instance age, level of education, socioeconomic status, ethnic background, physical attractiveness, intelligence, social attitudes, political orientation and personality variables [1]–[5], [12]–[13]. Nielsen and Svarer [14] indicate that next to age, education is the trait showing the highest degree of assortment. Accordingly, educational homogamy is a common phenomenon [2], [4], [10]–[11]. In the US, for example, one study reported that about 70% of marriages are educationally homogamous [11]. One reason for the high degree of assortative mating may lie in proximity effects, such as attending the same schools or sharing common work environments [6]. In addition, assortative mating may be advantageous in terms of increasing marital stability [6]–[8].

Our results show that assortative mating also carries reproductive advantages. We find a clear effect of educational homogamy on the chances to remain childless but not on the number of offspring. In each woman's educational category, the proportion of childless women was minimal or virtually minimal in those women married to a husband of equal educational category, whereas average offspring number was not significantly influenced by educational homogamy. Here, in line with Fieder and Huber [15], low educational attainment of both the woman and her husband increased mean offspring number. Average offspring number was thus only maximal in homogamously mated women of the lowest educational level.

Only little is known on the effects of assortative mating on reproductive success. Sporadic evidence exists that assortative mating enhances fertility as well as the number of surviving children [9]. As regards assortment for education, Bauer and Jacob [16] also find the highest odds becoming parents in educationally and occupationally homogamous couples. Mascie-Taylor [17] report decreasing fertility as educational homogamy decreases, and Bereczkei and Csanaky [6] find that women married to equally educated husbands have a reproductive success close to those married to higher educated husbands. Tsou et al. [18] showed that reproducing women married to a lower educated husband have fewer children than those married to an equally or higher educated husband, a pattern we did not find in our data which included childless individuals.

Even though we did not find a positive effect of educational homogamy on offspring number, it significantly lowered the odds of reproductive failure. We can only speculate why educational homogamy might decrease the chances of childlessness. Probably, it is not specifically educational homogamy that exerts an effect on the odds of childlessness, but assortative mating in general. We suggest that amongst other reasons, assortative mating may affect childlessness because of its effects on marital stability and satisfaction within the marriage. Couples facing high marital stability presumably rather decide becoming parents than those facing low marital stability. Lots of evidence show that similarity between partners benefits relationship satisfaction [19], marital stability [6]–[8], and earning [20], whereas heterogamous couples usually have a higher chance of dissatisfaction or divorce than homogamous ones [21]–[23]. The latter holds true particularly in educationally heterogamous couples where the wife is higher educated than the husband [24]. Divorce probability, however, is also lower if a least one spouse has a high educational attainment [25].

Another possible reason for the effects of educational homogamy on the odds of childlessness may be that homogamy has been shown to reduce stress levels in the partnership [26]. Preliminary analyses indicate that assortative mating even appears to be advantageous for a person's health estimation: we found that irrespective of a person's age, assortment for age in a marriage increased self estimation of general health as well as actual health indicators such as blood pressure (unpubl. data).

In our sample of couples still in their first marriage, educational homogamy was also associated with a lower average age at marriage. A later age at marriage may lead to a postponing of reproduction, which in turn is known to reduce reproductive output [27]. Hence, this finding might be still another reason for the reduced chances of childlessness in educationally homogamous couples. The lower mean age at marriage in homogamously married couples may be explained by the higher opportunity to meet a partner of similar education while still in school and university, respectively. Accordingly, completion of education and marriage often occur in fairly quick succession [28], typically resulting in a relatively young age at marriage. Though, Schwartz and Mare [29] find an inverted U shape age pattern of homogamy among new first marriages, with higher odds of educational homogamy among wives married between 26–29 year than among younger and older wives.

Educational heterogamy also appears to increase the time period between marriage and first birth. In a preliminary analysis of General Social Survey data from the US, the average period between marriage and first birth was tendencially longer in educationally heterogamous (2.2 years, n = 155) than in educationally homogamous couples (1.88 yr, n = 332; Mann-Whitney U-test: p = 0.059). A longer time period between marriage and first birth might thus also contribute to the effects of educational homogamy on the chances of childlessness.

To sum up, assortative mating based on educational level is a widespread phenomenon. It decreased the risk of childlessness but had no apparent effect on offspring number. We therefore conclude that educational homogamy lowers the odds of reproductive failure.
